# Long-term effects of pegvisomant on comorbidities in patients with acromegaly: a retrospective single-center study

**DOI:** 10.1530/EJE-15-0500

**Published:** 2015-11

**Authors:** Emmanuelle Kuhn, Luigi Maione, Amir Bouchachi, Myriam Rozière, Sylvie Salenave, Sylvie Brailly-Tabard, Jacques Young, Peter Kamenicky, Patrick Assayag, Philippe Chanson

**Affiliations:** 1 Assistance Publique-Hôpitaux de Paris, Hôpital de Bicêtre, Service d'Endocrinologie et des Maladies de la Reproduction, F-94275, Le Kremlin Bicêtre, France; 2 Service de Cardiologie, F-94275, Le Kremlin Bicêtre, France; 3 Service de Génétique moléculaire, Pharmacogénétique et Hormonologie, F-94275, Le Kremlin Bicêtre, France; 4 Univ Paris-Sud, Université Paris-Saclay, Faculté de Médecine Paris-Sud, Unité Mixte de Recherche-S1185, F-94276, Le Kremlin Bicêtre, France; 5 Institut National de la Santé et de la Recherche Médicale (INSERM) U1185, F-94276, Le Kremlin Bicêtre, France

## Abstract

**Context:**

The effect of pegvisomant on IGF1 levels in patients with acromegaly is well documented, but little is known of its long-term impact on comorbidity.

**Aim:**

The aim of this retrospective study was to evaluate the effects of long-term pegvisomant therapy on cardiorespiratory and metabolic comorbidity in patients with acromegaly.

**Patients and methods:**

We analyzed the long-term (up to 10 years) effect of pegvisomant therapy given alone (*n*=19, 45%) or in addition to somatostatin analogues and/or cabergoline (*n*=23, 55%) on echocardiographic, polysomnographic and metabolic parameters in respectively 42, 12 and 26 patients with acromegaly followed in Bicêtre hospital.

**Results:**

At the first cardiac evaluation, 20±16 months after pegvisomant introduction, IGF1 levels normalized in 29 (69%) of the 42 patients. The left ventricular ejection fraction (LVEF) improved significantly in patients whose basal LVEF was ≤60% and decreased in those whose LVEF was >70%. The left ventricular mass index (LVMi) decreased from 123±25 to 101±21 g/m^2^ (*P*<0.05) in the 17 patients with a basal LVMi higher than the median (91 g/m^2^), while it remained stable in the other patients. Pegvisomant reduced the apnoea–hypopnea index and cured obstructive sleep apnea (OSA) in four of the eight patients concerned. Long-term follow-up of 22 patients showed continuing improvements in cardiac parameters. The BMI and LDL cholesterol level increased minimally during pegvisomant therapy, and other lipid parameters were not modified.

**Conclusions:**

Long-term pegvisomant therapy not only normalizes IGF1 in a large proportion of patients but also improves cardiac and respiratory comorbidity.

## Introduction

Acromegaly is characterized by chronic growth hormone (GH) and insulin-like growth factor 1 (IGF1) hypersecretion and is associated with increased morbidity and mortality, notably due to cardiovascular and respiratory disorders (1, 2, 3). The specific cardiomyopathy of acromegaly consists of concentric biventricular hypertrophy, which can lead to diastolic and systolic dysfunction and to heart failure (2, 4, 5). These cardiac changes are due mainly to the GH and IGF1 excess, but the systemic arterial hypertension, glucose intolerance or diabetes, obesity and dyslipidemia frequently present in these patients are also likely to contribute to cardiovascular complications (6). Moreover, obstructive sleep apnea (OSA), which is present in most patients (7), is a risk factor for cardiovascular disorders and early death. Normalization of serum GH and IGF1 levels has been shown to improve clinical and metabolic abnormalities in acromegalic patients, especially cardiomyopathy, OSA, arterial hypertension and dyslipidemia (2, 7, 8, 9). However, biochemical control of acromegaly can be difficult to achieve, necessitating a multistep strategy (3). Surgical resection of GH-secreting pituitary adenomas cures some 50–70% of patients, depending on the surgeon's experience, the size and invasiveness of the tumor, and the degree of GH/IGF1 hypersecretion (10, 11). If surgery is unsuccessful, adjuvant medical therapies can reduce GH and IGF1 secretion. Long-acting somatostatin analogs (SAs) are generally tried first, and were shown in a recent meta-analysis to control GH and IGF1 levels in respectively 55 and 56% of patients (12). Cabergoline may also be used (13). When SAs, possibly combined with cabergoline, fail to control acromegaly, pegvisomant, a GH-receptor antagonist, normalizes serum IGF1 levels in 70–97% of cases (14, 15, 16, 17, 18, 19). Several studies have assessed the effect of pegvisomant on surrogate endpoints, but its impact on comorbidities of acromegaly (especially cardiomyopathy and OSA) is poorly documented. In a study of 12 patients with acromegaly, treatment with pegvisomant for 18 months improved cardiac hypertrophy and diastolic and systolic functions (20). In another study of 12 acromegalic patients, a 6-month course of pegvisomant reduced tongue volume and associated sleep disorders (21). As these studies were small and brief, we retrospectively evaluated the long-term effects pegvisomant on comorbidities among patients treated in our center, focusing on cardiac, respiratory and metabolic abnormalities.

## Patients and methods

This retrospective, single-centre study involved a cohort of 334 patients with acromegaly who were regularly followed at Bicêtre University Hospital since 1993. Eighty of these patients received pegvisomant at some time between 2003 and 2013. This study focused on 42 patients with available echocardiographic findings prior to pegvisomant introduction and who received this drug for at least 3 months before undergoing repeat echocardiography. The mean duration of pegvisomant therapy in these 42 patients at the first evaluation after pegvisomant introduction was 20±16 months (3–60). The other 38 patients were excluded because of either shorter treatment or unavailable or poor-quality echocardiographic data. The main characteristics of this latter group were similar in terms of sex ratio, age at diagnosis, size of the macroadenoma, time between diagnosis of acromegaly and initiation of pegvisomant, previous treatments, metabolic parameters (except for use of statins, 5% in patients included vs 15% in patients not included, *P*=0.03), hypertension, IGF1 levels, type of treatment (pegvisomant alone or in association). The left ventricular ejection fraction (LVEF) was measured at least once in 32 of the 42 patients, and left ventricular mass indexed to the body surface area (LVMi) was determined at least once in 35 patients. Serial metabolic analyses were available for 26 patients. Serial cardiac evaluations were available for 22 patients, allowing us to study improvements in cardiac parameters (LVEF and LVMi in 16 and 19 patients respectively) associated with IGF1 normalization on pegvisomant. A third cardiac evaluation was available for nine patients.

Twelve patients who underwent polysomnography before and during pegvisomant therapy were also studied for the effect of pegvisomant on OSA.

The main characteristics of the 42 patients included in the study are summarized in Table 1. The adenoma was purely somatotroph (81%) or mixed somato-lactotroph (19%). Pegvisomant was prescribed following failure or intolerance of other drugs (SAs or dopamine agonist), either alone (*n*=19, 45%) or in combination with other treatments (*n*=23, 55%).

Serum IGF1 was measured with a commercial kit (22). Total cholesterol (TC), HDL cholesterol (HDLc) and triglyceride (TG) levels were measured before pegvisomant introduction, as soon as serum IGF1 level normalized and at the time of last available IGF1 determination. LDL cholesterol (LDLc) levels were calculated with Friedewald's formula.

Regular echocardiography is a routine element of acromegalic patient management in our center. Most examinations analyzed here were performed in the Bicêtre Cardiology Department using a General Electrics Vivid 7 system (GE Medical Systems, Milwaukee, WI, USA). All evaluations were made by experienced cardiologists and included two-dimensional Doppler and color Doppler scans as previously described (23). LVMi was calculated with Devereux's formula (24). Left ventricular hypertrophy (LVH) was defined by LVMi >135 g/m^2^ in male and >110 g/m^2^ in female patients (24).

Polysomnographic data were recorded with a 16-channel polygraph in the same center, as previously described (25). Briefly, apnea was defined as the cessation of oronasal airflow for more than 10 s. Apnea was considered obstructive when there was evidence of persistent respiratory effort. Hypopnea was defined as a reduction in oronasal airflow to at least 50% of the value prevailing during normal breathing, for at least 10 s, followed by transient electro-encephalographic arousals, or as a smaller reduction in airflow associated with ≥4% desaturation. The American Academy of Sleep Medicine defines OSA as ≥15 events/h with or without OSA symptoms, or as ≥5 events/h with OSA symptoms. OSA severity is classified according to apnea–hypopnea index (AHI) and is defined as mild if 5–14 events/h, moderate if 15–30 events/h, and severe if >30 events/h (26, 27).

### Statistical analysis

Data are reported as mean±s.d., mean and range, or percentages as appropriate. Paired analyses were performed using the non-parametric Wilcoxon test or one-way ANOVA Friedman's test with Prism 5 software (Graph-Pad Software, San Diego, CA, USA). *P* values <0.05 were considered to denote statistical significance.

## Results

### Long-term cardiac outcome on pegvisomant

#### In the whole group of patients

Baseline cardiac evaluation was performed 6±9 months before starting treatment with pegvisomant (41 of these 42 patients had already received a treatment – surgery, radiotherapy, SAs and/or cabergoline – for acromegaly before this basal evaluation) and follow-up echocardiographic evaluation was carried out after 20±16 months of pegvisomant therapy.

**Figure 1:**
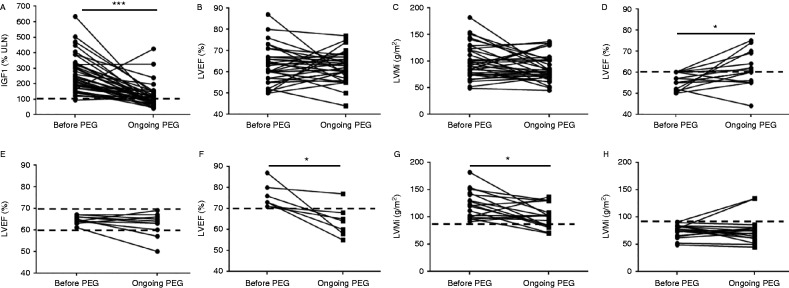
Effect of pegvisomant (PEG) on cardiac structure. Upper panels: individual values of IGF1 (A, *n*=42), left ventricular ejection fraction (LVEF) (B, *n*=32) and left ventricular mass index (LVMi) (C, *n*=35) before pegvisomant and at the first on-treatment evaluation. Middle panels: changes in LVEF as a function of baseline values (D: LVEF <60%, E: LVEF 60–70%, F: LVEF >70%). Lower panels: changes in LVMi as a function of baseline values (G: LVMi above median baseline value, H: LVMi below median baseline value). Statistical significance: *P<0.05, ***P<0.001.

On a mean daily pegvisomant dose of 25±11 mg/day, serum IGF1 levels (Fig. 1A) fell from 271±125% to 113±73% of the upper limit of the normal range (ULN) (*P*<0.0001). Overall, the LVEF (*n*=32, Fig. 1B) did not change significantly. However, when patients were subdivided according to their basal LVEF (≤60% or >70%), a significant increase (from 56±4% to 61±8%, *P*<0.05) was observed in the subgroup of patients with initial LVEF ≤60% (Fig. 1D), while LEVF fell from 76±6% to 64±7% (*P*<0.05) in the subgroup of patients with initial LVEF >70% (Fig. 1F). LVEF did not change in the subgroup of patients with baseline values between 60 and 70% (Fig. 1E).

Overall, the LVMi (*n*=35, Fig. 1C) did not change significantly. However, prevalence of LVH decreased from 20% (seven patients out of 35) to 11% (four patients out of 35) and in the subgroup of 17 patients with a baseline LVMi above the median (91 g/m^2^), LVMi fell significantly during pegvisomant treatment from 123±25 to 101±21 g/m^2^ (*P*<0.05, Fig. 1G). In contrast, LVMi did not change significantly in the 18 patients whose baseline LVMi was below the median (75±12 vs 76±24 g/m^2^, Fig. 1H).

#### In patients whose IGF1 normalized on pegvisomant

**Figure 2:**
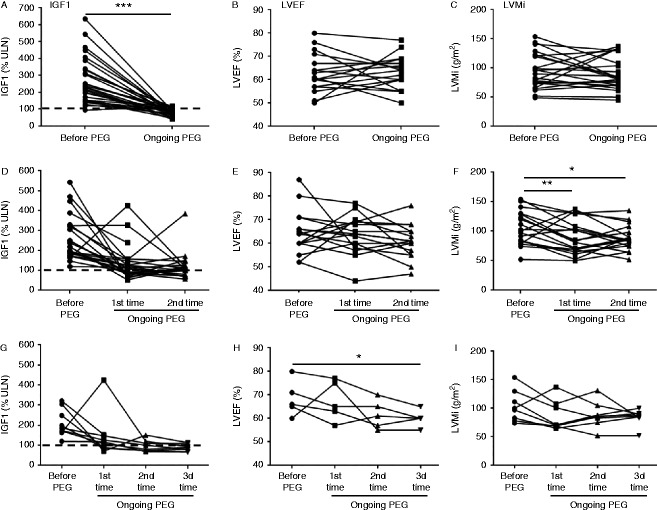
Long-term effects of pegvisomant (PEG) on cardiac parameters in patients with controlled acromegaly (IGF1 <115% ULN). Upper panels: individual values of IGF1 (A), left ventricular ejection fraction (LVEF) (B) and left ventricular mass index (LVMi) (C) in 29 patients before pegvisomant and at the first on-treatment evaluation. Middle panels: individual values of IGF1 (D, *n*=23), LVEF (E, *n*=16) and LVMi (F, *n*=19) before pegvisomant and at the first and second on-treatment evaluations. Lower panel: individual values of IGF1 (G), LVEF (H) and LVMi (I) in nine patients before pegvisomant and at the first, second and third on-treatment evaluations. Statistical significance: *P<0.05, **P<0.01, ***P<0.001.

At the first evaluation during pegvisomant therapy (20±16 months), serum IGF1 levels had achieved normal or near-normal levels (<115% of ULN) in 29 (69%) of the 42 patients (Fig. 2A). As compared with baseline values, no overall change in LVEF was observed (63±8 vs 63±7%, Fig. 2B), while LVMi tended to decrease during pegvisomant treatment (97±34 vs 89±27 kg/m^2^) in patients whose IGF1 levels normalized.

#### In patients whose IGF1 levels did not normalize

LVMi also tended to decrease in the ten patients whose serum IGF1 levels, although decreased, did not achieve normal or near-normal levels on pegvisomant (98±26 vs 85±24%, *P*=0.06).

### Results of serial echocardiography

Serial echocardiographic findings (two or more procedures) were available for 22 patients, of whom nine had a third cardiac evaluation.

Baseline echocardiography took place 8±11 months before pegvisomant introduction, and the first, second and third on-treatment cardiac evaluations were performed 20±15, 54±28 and 76±30 months after pegvisomant introduction in respectively 42, 23 and nine patients. Serum IGF1 levels fell from 265±114% ULN at baseline to 138±88% at the first evaluation, on an average dose of 25 (10–40) mg/day, then to 119±70% ULN at the second evaluation, on an average dose of 33 (15–80) mg/day (Fig. 2D), and 87±17% ULN at the third evaluation, on an average dose of 37 (15–80) mg/day (Fig. 2G).

LVEF did not change significantly during treatment (Fig. 2E), whereas LVMi fell by 14% at the first evaluation (from 104±28 to 90±28 g/m^2^, *P*<0.01) and by 16% at the second evaluation (from 104±28 to 88±20 g/m^2^, *P*<0.05) (Fig. 2F). The prevalence of LVH fell from 22% (four out of 18 patients) to 6% (one out of 18) at the first evaluation and to 0% at the second evaluation. At the third evaluation, LVEF (available in five patients) continued to decrease significantly (*P*<0.05) (Fig. 2H), whereas LVMi (available in eight patients) showed only a trend towards lower values (Fig. 2I). The only patient with LVH reverted it.

During this follow up, mean systolic and diastolic blood pressure did not change significantly, neither in patients who were normotensive nor in those who were hypertensive at baseline. Indeed, in normotensive patients, mean systolic/diastolic blood pressure was 126/74 mmHg (vs 124/69 mmHg before pegvisomant) at first evaluation under pegvisomant (*n*=22), and 123/81 mmHg (vs 127/70 mmHg) at the second evaluation under pegvisomant (*n*=11). In hypertensive patients, mean blood pressure was 135/78 mmHg (vs 130/77 mmHg) at first evaluation under pegvisomant (*n*=10) and 143/82 mmHg (vs 129/79 mmHg) at the second evaluation under pegvisomant (*n*=6). Similarly the number of anti-hypertensive drugs received by hypertensive patients remained the same.

### Effect of pegvisomant on OSA

**Figure 3:**
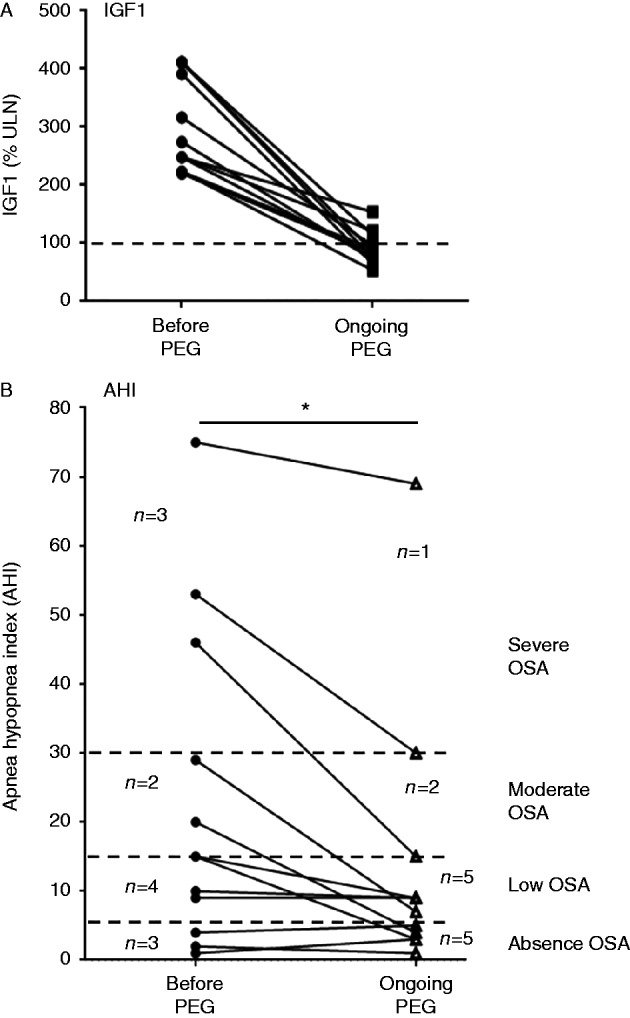
Effect of pegvisomant (PEG) on the apnea–hypopnea index (AHI) in 12 patients. Upper panel (A): individual IGF1 values in 12 patients before and during treatment with pegvisomant. IGF1 normalized (<115% ULN) in 10/12 patients (83%). Lower panel (B): changes in AHI on pegvisomant. Dotted lines represent the different stages of obstructive sleep apnea (OSA). Before pegvisomant: severe OSA (AHI >30/h, *n*=3), moderate OSA (15<AHI≤30/h, *n*=2), mild OSA (5<AHI≤15/h, *n*=4), no OSA (AHI≤5/h, *n*=3). On pegvisomant: severe OSA (*n*=1), moderate OSA (*n*=2), mild OSA (*n*=5), no OSA (*n*=5). Statistical significance: *P<0.05 (before pegvisomant vs on pegvisomant).

The impact of pegvisomant on OSA, defined by an AHI higher than 5/h on polysomnography, was evaluated after 16±17 months (3–64) of treatment in a subgroup of 12 patients. As shown in Fig. 3A, the mean serum IGF1 level fell from 301±81% to 89±28% ULN (*P*<0.0001) in these 12 patients, who took 10–40 mg/day pegvisomant. IGF1 normalized in ten patients (83%). Overall, AHI showed a statistically significant improvement (*P*<0.05). At baseline, OSA was severe (AHI>30/h), moderate (15<AHI≤30/h) and mild (5<AHI≤15/h) in three, two and four patients, respectively, while three patients did not have OSA (AHI≤5/h). On pegvisomant, repeat polysomnography showed severe, moderate and mild OSA in respectively one, two and five patients, while five patients did not have OSA. OSA (>10/h) improved in six of eight patients and disappeared in four patients.

### Effect of pegvisomant on BMI and metabolic parameters

To evaluate the long-term metabolic effects of pegvisomant, we collected data on the BMI and lipid and glucose metabolism in 25 patients.

BMI rose from 31±5 kg/m^2^ at baseline to 32±7 kg/m^2^ (*P*=0.02) at the last on-treatment evaluation. However, this increase in BMI had already occurred before pegvisomant introduction, following the reduction in IGF1 levels due to previous treatments (surgery, SAs, etc.). Indeed, these 25 patients' mean BMI was 29±4 kg/m^2^ at diagnosis of acromegaly, 31±5 kg/m^2^ before pegvisomant introduction (*P*=0.003 vs BMI at diagnosis), 33±7 kg/m^2^ when serum IGF1 first normalized (after 15±15 months on pegvisomant, *P*=0.0559 vs BMI before pegvisomant) and finally to 32±7 kg/m^2^ at the last assessment (after 58±21 months on pegvisomant, *P*=0.3 vs BMI before pegvisomant). At this last assessment, the serum IGF1 level was normal in 70% of patients.

**Figure 4:**
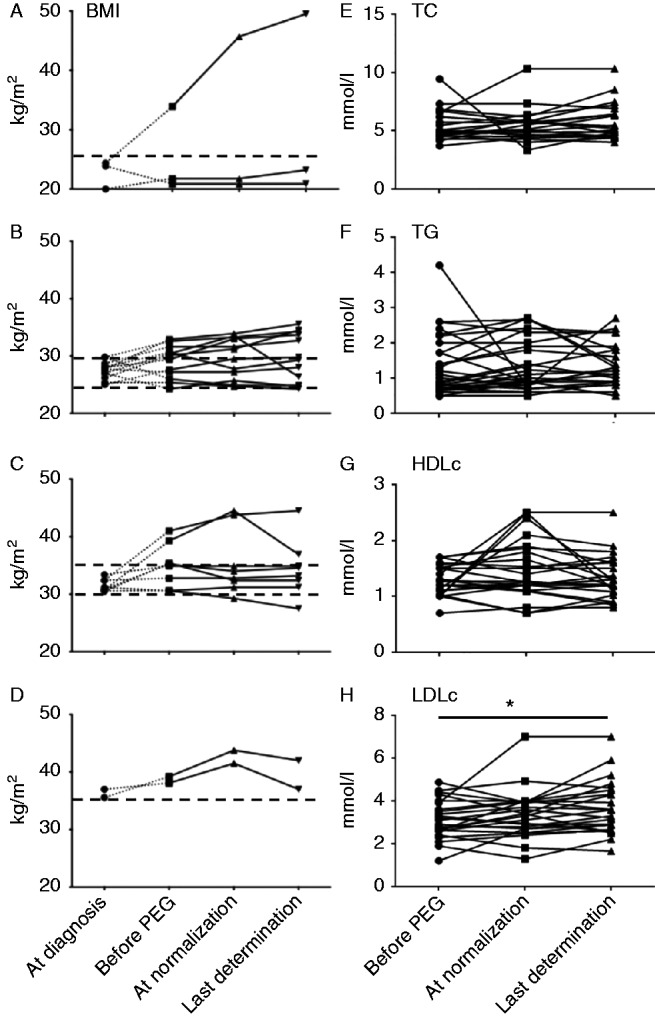
Changes in BMI and lipid parameters on pegvisomant (PEG). Left panels: changes in BMI on pegvisomant. Individual values are given as a function of BMI at diagnosis: BMI<25 kg/m^2^ (A), 25≤BMI<30 kg/m^2^ (B), 30≤BMI<35 kg/m^2^ (C), BMI≥35 kg/m^2^ (D). Right-hand panels: changes in plasma lipid levels on pegvisomant. Individual values of total cholesterol (TC, E), triglycerides (TG, F), HDL cholesterol (HDLc, G) and LDL cholesterol (LDLc, H) are shown. *P<0.05.

Figure 4 shows individual changes in BMI on pegvisomant according to the BMI category at diagnosis of acromegaly. In the three patients whose BMI at diagnosis was <25 kg/m^2^, BMI increased markedly in one case and did not change in the other two cases (Fig. 4A). BMI rose gradually during pegvisomant treatment in seven of the 12 patients who were overweight at diagnosis (BMI ≥25 but <30 kg/m^2^). This rise in BMI started after the first treatment for acromegaly (Fig. 4B). The slope of weight gain was the same before and after treatment with pegvisomant and ran parallel to the gradual improvement in IGF1 levels, whatever the treatment. Similar results were obtained in six of the eight obese patients (BMI >30 kg/m^2^) (Fig. 4C) and in the two patients with morbid obesity (BMI >35 kg/m^2^) (Fig. 4D).

Long-term changes in plasma lipid levels during pegvisomant treatment were studied in patients who did not receive statins or fibrates, and were measured at the same time as BMI. As shown in Fig. 4E and G, TC, TG and HDLc plasma levels were not modified by long-term pegvisomant therapy. In contrast, the plasma LDLc level rose gradually from 3.1±0.9 mmol/l before pegvisomant to 3.7±1.2 mmol/l at the last measurement (*P*<0.05) (Fig. 4H).

In 20 patients who were free of diabetes before pegvisomant introduction, the mean fasting blood glucose level did not change on pegvisomant: their values were 5.4±1.4, 5.1±0.9 and 5.6±1.8 mmol/l, respectively, before pegvisomant, at IGF1 normalization, and at the last available determination (NS). In diabetic patients, pegvisomant had no influence on FBG, HbA1c or the number of anti-diabetic drugs received, probably owing to the variable length of follow-up, co-administration of SAs in some patients, and changes in anti-diabetic drug regimens (data not shown).

## Discussion

This clinical audit shows that long-term treatment with pegvisomant (up to 10 years) is associated with gradual improvements in cardiac and respiratory comorbidity associated with acromegaly, in parallel with the improvement in IGF1 levels. Systolic dysfunction (LVEF <60%) improved on pegvisomant, and LVEF normalized in patients with a hyperkinetic syndrome (LVEF >70%). LV hypertrophy also improved on pegvisomant, as shown by the decrease in LVMi in patients with the most severe LV hypertrophy. Pegvisomant also improved OSA, with a reduction in the AHI in most of the patients concerned. Finally, BMI and LDLc increased on pegvisomant but other lipid parameters did not change.

While studies of the effect of pegvisomant on surrogate outcomes such as the IGF1 level are numerous, the impact of this GH receptor antagonist on comorbidities of acromegaly is poorly documented, despite health authorities' growing demands for hard endpoints. Our study is the first to show the effects of pegvisomant on severe comorbidities of acromegaly in real-life conditions.

Patients with untreated or uncontrolled acromegaly are at risk of cardiomyopathy, a major comorbidity. Acromegalic cardiomyopathy includes early LVH with a hyperkinetic syndrome and subsequent diastolic and systolic dysfunction. This cardiomyopathy is due to both a direct action of GH/IGF1 on the heart and to cardiovascular risk factors such as arterial hypertension and glucose intolerance (2, 4, 6). The cardiac hypertrophy observed by echocardiography or MRI includes cardiac muscle hypertrophy related to the direct effect of GH on muscle (28) and to the effect of frequently associated hypertension (29), but also to water infiltration of the myocardium, as demonstrated by the T2 relaxation time on cardiac MRI (30). Water infiltration observed in acromegaly (31) is secondary to increased sodium reabsorption due to overexpression of the epithelial sodium channel (ENaC) in distal tubules (32, 33). By contrast with diabetes mellitus, where insulin resistance is associated with myocardial fat deposition (contributing to cardiac hypertrophy and dysfunction), a recent cardiac MRI study of patients with acromegaly showed that, despite insulin resistance, the lipid content of both the heart and liver was reduced (34). Acromegalic cardiomyopathy can be slowed or prevented by suppressing GH and IGF1 oversecretion (2, 8, 35). SAs improve acromegalic cardiomyopathy (8, 36) but, although well tolerated, are effective in only about 55% of acromegalic patients according to a recent meta-analysis (12). Pegvisomant acts by blocking GH receptor dimerization, leading to a fall in IGF1 synthesis. In surveillance studies of pegvisomant therapy, IGF1 normalization is achieved in 60–70% of patients (3, 19). Unlike SAs, which inhibit not only GH but also insulin secretion, pegvisomant normalizes IGF1 levels in most patients with acromegaly and has beneficial effects on glucose metabolism because it does not modify insulin secretion (37).

All previous studies of the effect of pegvisomant on cardiac structure, OSA and metabolic parameters involved small groups of patients and lasted no more than 18 months (reviewed in (7, 8, 38)). Ours is the first study to analyze the impact of pegvisomant on these different parameters during long-term treatment (up to 10 years) in a large number of routinely managed patients. We show that pegvisomant can reduce LVMi, an effect that persists and sometimes continues to improve with time. This is in keeping with the results of a prospective study of 17 patients in whom LVMi was significantly reduced after 18 months on pegvisomant (20). The improvement observed here is all the more noteworthy because pre-pegvisomant echocardiography was generally performed after patients had already received treatment with surgery and/or SAs or dopamine agonists, even though GH/IGF1 levels had failed to normalize. LVMi and LVEF improved on pegvisomant even when IGF1 levels had not normalized by the first on-treatment evaluation. This underlines the beneficial effect on cardiomyopathy of time elapsed with normal (or even only reduced) IGF1 levels. We did not study the effect of pegvisomant on cardiac arrhythmias, but the observed improvement in cardiac morphology and function is likely to be beneficial, as observed in a previous study in which these parameters were monitored during 18 months of pegvisomant therapy (39). During the follow-up systolic and diastolic blood pressure as well as the number of antihypertensive drugs number were not modified by pegvisomant suggesting that the effect of hypertension and its treatment is likely to be of minor importance in the improvement of cardiac parameters observed during treatment with pegvisomant.

Patients with acromegaly have an increased risk of OSA, which is attributed to soft-tissue hypertrophy in the upper airways, maxillo-facial bone changes and, possibly, pharyngeal dilator muscle impairment (7). Treatment of acromegaly has a favorable effect on OSA, but about 40% of patients in whom acromegaly is controlled still have AOS (25, 40, 41, 42). In the present study, OSA was improved by pegvisomant, as shown by the decrease in the mean AHI of the whole group and in the number of patients with severe OSA. These results are in keeping with those of the only other study to evaluate the influence of acromegalic disease control by pegvisomant on OSA: a 6-month course of this drug in 12 patients with acromegaly was associated with reduced tongue volume and AHI (21). Again, the improvement on pegvisomant is all the more noteworthy in view of the patients' previous treatments for acromegaly, which could already have improved their OSA status.

Long-term treatment with pegvisomant was associated with a trend towards an increase in BMI and with a significant rise in the plasma LDLc level. Data on changes in BMI during treatment for acromegaly, particularly with pegvisomant, are scarce and conflicting: some studies have shown an increase in BMI (43, 44) while others showed no change (9, 45, 46). Overall, however, the data suggest an increase in fat mass (47, 48), particularly intra-abdominal fat mass (49), and also a decrease in lean body mass and extracellular water, which might explain the neutral effect of pegvisomant on BMI in some studies.

Pegvisomant had no effect on TC, TG or HDL-c levels, while LDLc increased. These results are consistent with those of two previous studies in which pegvisomant was associated with an increase in total and LDLc (50, 51), although another study showed no effect (52). Long-term treatment with pegvisomant had no influence on fasting blood glucose levels in our non diabetic patients. While pegvisomant monotherapy is known to improve glucose metabolism (17, 53), particularly in patients having switched from a SA (9, 37, 54), we observed no effect on glucose metabolism, probably because of interference by SA co-administration or changes in antidiabetic drug regimens.

The main limitation of our study is its retrospective design and the resulting large number of missing data, particularly because the study was performed in real-life conditions and reflected our local clinical practice. Another limitation of the study is the low number of patients included in some subgroups which may induce some bias in interpreting the results. For example, effects on LVEF associated with pegvisomant treatment could simply be related to regression towards the mean. Lastly, the place of pegvisomant, compared to other treatments of acromegaly, particularly when pegvisomant is associated with other treatments, is difficult to assess. We reviewed data from our patients controlled by surgery alone or by somatostatin analogues alone and in whom serial echocardiographic studies with complete data before treatment and after IGF1 normalization were available and did not find differences in terms of BMI, blood pressure and cardiac parameters between both periods (data not shown). However, the low number (six patients controlled by surgery and six patients controlled by SAs alone) precludes any firm conclusion. Thus large and long-term prospective studies performed in acromegalic patients are required to assess precisely the impact of pegvisomant treatment on acromegaly comorbidities and to compare this effect with that achieved with other types of treatment (surgery or somatostatin analogues).

In conclusion, this retrospective single-center study shows that long-term treatment of acromegalic patients with pegvisomant in routine clinical practice improves not only surrogate endpoints such as the IGF1 level but also comorbidities. Long-term follow-up with serial cardiac evaluation showed a continuous improvement in cardiomyopathy. We also confirm that pegvisomant improves sleep apnea, curing it in half the patients concerned. Pegvisomant was associated with an increase in BMI, which had already started to increase after previous treatments for acromegaly; bodyweight changes thus seem to reflect the improvement in IGF1 levels. The increase in LDLc levels on pegvisomant was minimal. Large prospective studies are now needed to assess the impact of treatments for acromegaly on associated comorbidities.

## Figures and Tables

**Table 1:** Main characteristics of the 42 patients included in this retrospective study of the effects of long-term treatment with pegvisomant on acromegalic comorbidities.

	
Number, *n*	42
Males/females	26/16
Age at diagnosis, years (range)	36 (15–64)
Macroadenoma, *n* (%)	40 (95)
Time between diagnosis of acromegaly and pegvisomant introduction (months)	55 (3–240)
Treatments prior to pegvisomant	
Surgery, *n*	32
Radiotherapy, *n*	7
Somatostatin analogs, *n*	40
Cabergoline, *n*	21
Metabolic parameters at pegvisomant introduction	
BMI (kg/m^2^), mean (range)	31 (21–41)
Diabetes, *n* (%)	10 (24)
Statin therapy, *n* (%)	2 (5)
Hypertension at pegvisomant introduction, *n* (%)	11 (26)
Mean IGF1 (% ULN) at pegvisomant introduction (range)	271 (94–633)
Treatment	
Pegvisomant alone, *n* (%)	19 (45)
Pegvisomant plus other drugs, *n* (%)	23 (55)
Somatostatin analogs, *n*	15
Cabergoline, *n*	15
